# Online Interviews as New Methodological Normalcy and a Space of Ethics: An Autoethnographic Investigation into Covid-19 Educational Research

**DOI:** 10.1177/10778004231176283

**Published:** 2023-05-26

**Authors:** Hongming Fan, Bingqing Li, Truly Pasaribu, Raqib Chowdhury

**Affiliations:** 1 Monash University, Melbourne, Victoria, Australia; 2 La Trobe University, Melbourne, Victoria, Australia; 3 Universitas Sanata Dharma, Yogyakarta, Indonesia

**Keywords:** Covid-19 pandemic, online interviews, early-career researchers, empowerment, ethics in practice, autoethnography

## Abstract

Worldwide travel restrictions during the Covid-19 pandemic abruptly changed the norms of conducting qualitative research. Online interviews, long regarded as a second choice to their offline counterparts, are no longer seen as supplementary since they emerged as the dominant mode of data collection during the pandemic. This study employs an autoethnographic approach to investigate the authors’ experiences of adjusting to alternative methodological approaches. The investigation critically reflects on how the author’s agencies in allocating and gathering instructional, social, and economic resources led to a researcher identity reconfigured by choices in making ethical commitment in data collection. This article also sheds light on how the authors, constrained by limited resources, gained better understanding of ethics in practice through negotiation with participants and obtained rich data by exercising their agencies. The article argues that researchers need to place both online and offline methods on equal footing to facilitate a more ethically sensitive approach to data collection.

## Introduction

Worldwide travel restrictions during the Covid-19 pandemic abruptly changed the norms of conducting qualitative research. While there is less doubt that the widespread use of the internet has developed and reshaped qualitative research technologically, socially, and culturally (Bryman, 2016; Fischer et al., 2017) over the last two decades, literature suggests that many researchers still regard online data collection as a second choice to offline data collection involving interviews (Abrams & Gaiser, 2017; Deakin & Wakefield, 2014). Such perspectives remained prevalent until the outbreak of the Covid-19 pandemic in early 2020 when methods such as online interviews became no longer a second choice but in many cases the only mode of collecting data due to physical distancing and worldwide travel restrictions.

Although some studies have highlighted readjustments in the practicalities of preparing and conducting online interviews during the pandemic (e.g., Howlett, 2021; Lawrence, 2022), less attention has been paid to researchers’ lived experiences in making ethical adaptations while being forced to transition from offline to online interviews. As Howlett (2021) notes, due to the prevalence of the former in social sciences research, many researchers had not considered, received training, or experienced online interviews before the pandemic. This would make it challenging for them to adjust to the new social configurations of the Covid-19 pandemic, particularly for early-career researchers (ECRs; e.g., PhD students), who are less experienced and still formulating their researcher identities. In addition, while transiting to online data collection, maintaining the ethics and quality of research while gathering rich data presented new challenges that needed to be understood to facilitate the futureproofing of such methods. In this article, we argue that such a transition indelibly affects ECRs, often leaving a lingering influence on their understanding of doing research ethically. Considering the far-reaching and long-lasting impacts of the social and economic ruptures which have arisen in the aftermath of the pandemic (Li, 2022), it is thus important to better understand the influence of such changes in the agentic role of ECRs.

In this article, three PhD researchers reflect how a set of complex adjustments from offline interviews to online interviews prompted by the pandemic shaped their understanding of doing qualitative research and how such adjustments helped them engage with participants in more ethically sensitive ways. The study particularly focuses on online experiences of conducting individual and group interviews. We believe shedding light on such issues would not only assist a better understanding of ECRs’ development of their academic researcher identity, but also contribute to a new understanding of research ethics in the foreseeable digital future (Bounfour, 2016). To provide insights into future trends that we are likely to see in qualitative research, this article aims to explore what challenges and forms of negotiation researchers need to engage in when adapting to online interviews and how this experience shapes researchers’ understanding of doing online research ethically.

With these aims, after summarizing the literature on online data collection, we rationalize the choice of empowerment theory and justify the selection of autoethnography to scrutinize the complexities of our lived experiences as researchers in collecting data through online interviews. Finally, we highlight contextual reflections for future researchers who are considering adopting online data collection for their qualitative research. The findings and discussion are expected to provide further methodological guides for researchers who are conducting online research.

## Literature Review

Before framing the narratives using Kabeer’s (1999a) theory of empowerment, it is of critical importance to understand how online interview as a method has been conceptualized in the literature. Depending on the approaches adopted for conducting interviews, there are three distinct categories: online or offline interviews based on the technology used; synchronous or asynchronous interviews according to speed of reply; text-based or oral interviews which could be offline, audio, or video interviews (Abrams & Gaiser, 2017; O’Connor & Madge, 2017). As the authors conducted both online video and offline oral data collection, this study would concentrate on the distinction of such modes.

While for many years some online data collection methods (e.g., online surveys) have been used as the mainstream mode in social science studies, with interviews, most researchers tacitly regarded it as an offline method before the Covid-19 pandemic (Abrams & Gaiser, 2017; O’Connor & Madge, 2017). In fact, online interviews were seen as a “second choice” when offline communication was not available (Deakin & Wakefield, 2014, p. 604). Previous research comparing online interviews with their offline counterparts such as those conducted by Bryman (2016) and Creswell and Poth (2018) point to the online methods’ strengths, including their cost efficiency and logistic ease, especially with dispersed or inaccessible people, as well as their weaknesses, such as the difficulty in building up rapport and recruiting participants. Despite these differences, Thunberg and Arnell (2021), writing during the pandemic, argue that there is little difference between online and offline methods both in terms of data quantity and quality. This is supported by an earlier, pre-pandemic study by Woodyatt et al. (2016) who observe that the content of the data collected from online and offline group interviews are highly similar, although the data formats may vary. Therefore, Creswell and Poth (2018) encouraged researchers to employ online interviews where and when possible and advised editors and readers to consider such studies. Largely, such recommendations were met with skepticism from researchers until the Covid-19 pandemic.

The outbreak of the pandemic in 2020 triggered renewed attention to online interviews when it became no longer a second choice or supplementation but the only option as collecting data in person became impossible for many researchers due to worldwide travel restrictions and the lack of research funding. However, online research methods are not the same as offline methods, thereby requiring thoughtful considerations to identify probable methodological and ethical challenges (Newman et al., 2021). As mentioned above, however, few researchers were equipped with enough knowledge and skills, and thus confidence, in online interviews before the pandemic (Howlett, 2021). Such lack of knowledge and skills disempowered researchers by making them feel bewildered and uncertain about how to collect data in such an emergent situation. This was especially true for ECRs who had relatively less or no experience. Since then, researchers have sought to learn and discuss the design and implementation of online interviews in order to empower themselves (e.g., Heiselberg & Stępińska, 2022; Lawrence, 2022; Wray & Barrett, 2022; Żadkowska et al., 2022).

Yet, thus far, very few studies have explored the challenges and struggles that researchers experienced when adopting online interviews during the pandemic. Arguably, such experiences would have a continuing impact on our perspectives of conducting research in the post-pandemic period. This article attempts to shed light on how these ECRs with limited resources during Covid-19 pandemic obtained rich data by exercising their agency and gained better understanding of doing online interviews ethically.

This manner of negotiating alternative methodological choices can be understood as a newly emerging aspect of “ethics in practice” (Guillemin & Gillam, 2004, p. 261). Ethics in practice, which involves everyday ethical issues arising in doing research, is an important dimension of research ethics (Guillemin & Gillam, 2004). Another dimension of research ethics is “procedural ethics,” which refers to seeking approval from an ethics committee and following their codes and guidelines (Guillemin & Gillam, 2004, p. 261). However, as argued by many researchers (e.g., Guillemin & Gillam, 2004; Sugiura et al., 2017), organizations’ codes of conduct or general guidelines can be insufficient in coping with ethical questions at the local or individual levels, or in cross-cultural settings, which necessitated ethics in practice (Guillemin & Gillam, 2004; Roberts, 2015). It involves many immediate ethical concerns that researchers need to consider to respond to the specific task of data collection. Although offline interviews have gradually resumed with the easing of restrictions in the post-pandemic times, online interviews have gained increasing acceptance since some researchers and participants prefer online methods as the findings of this article suggest. In this case, researchers and participants need to co-negotiate the selection of interview approaches, which serves as a new aspect of ethics in practice. Having freedom in making such selections also empower researchers by demonstrating the awareness of being in a certain manner of “acting visible to others” (Foucault, 1984, p. 117). That said, ethical principles still guide researchers to be open to their participants in terms of their research positions, platforms, and procedures to provide choices to participants to voice out their lived experiences.

When providing such choice, however, researchers need to consider possible ethical issues, such as different ways to engage with and respond to participants in online and offline interviews. As Sugiura et al. (2017) claim, online research has created new challenges and forced researchers to rethink existing ethical principles. This means researchers may be confronted with some “ethically important moments” that they have not encountered in offline interviews (Guillemin & Gillam, 2004, p. 261). Ethically important moments refer to subtle and often unpredictable situations which occur during the implementation of research (Guillemin & Gillam, 2004). Researchers need to exert their agency to cope with such situations. After all, the authors’ research experience of online interviews is not only a methodological change but involves a new configuration of ethical considerations. To better understand such changes and considerations, it is necessary to investigate how the researchers exercise their agency to assess and use the resources available to them during the pandemic, complete online interviews, then reshape their understanding of doing research and negotiating the selection of data collection methods with the participants, ideally leading to mutual benefit and respect.

## Theoretical Framework: Kabeer’s Empowerment Theory

In this study, we adopted Kabeer’s empowerment theory (Kabeer, 1999a, 1999b, 2005) as a lens to problematize and understand the multidimensionality of three ECRs’ lived experiences of adapting digital technologies into interviews during and after the Covid-19 pandemic. According to Kabeer (1999b), empowerment involves “a process of change” (p. 437), obtaining more opportunities for exercising choices in life (in this case, the choice of online rather than face to face interviews) from a somehow disadvantaged place (in this case, the Covid-19 pandemic). Although initially applied in research investigating women’s empowerment, Kabeer’s framework provides an efficient perspective to look at how ECRs coped with the situation during a global health crisis when taken-for-granted research procedures were broadly unavailable, and more importantly, how their agentic movements had altered their academic identities. Particularly, the three interrelated dimensions of Kabeer’s empowerment theory—resources, agency, and achievement—lead the critical reflections and discussions in this study.

Kabeer (1999a) explains that, as the preconditional aspect of empowerment, material, human, and social resources enhance a person’s ability to make choices. For researchers, material resources may include funding, devices and access to the internet and digital applications. Human and social resources, which are equally important, may take the forms of access to the field, instructions, research experiences, and researchers’ relationships with participants. There is a special form of social resource which is rules and norms that “demarcate the boundaries of choice” (Kabeer, 1999a, p. 3). In academia, the prevailing tacit acknowledgment that interviews should be implemented in person made online interviews a less preferable option (Howlett, 2021). However, under the conditions of the pandemic, while many resources for offline interviews were limited, thereby disempowering researchers in making choices, the authors had to seek alternative modes for online interviews to conduct research. In this sense, the utilization of available resources becomes the basis for an actor (the researcher) to practice their agency (Kabeer, 2005).

The second dimension of empowerment is related to individual and collective agency. According to Kabeer (1999a, 1999b, 2005), agency refers to the process of defining and acting upon goals. She believes that practising agency involves not only observable actions, such as decision-making and change-making, but also the meaning that an individual brings in their behaviors or “power within” (p. 438). Emirbayer and Mische (1998) further explain that human agency requires actors’ engagement in different structural environments in which they negotiate through “habit, imagination and judgement, both reproduces and transforms those structures in interactive response to the problems posed by changing historical situations” (p. 970). That is to say, agency incorporates different temporalities, positioning actors’ continual reconstruction of their current orientations in the reflections on the past and future responses to emerging situations (Chakma, 2023).

In the case of this study, the authors’ agencies were manifested through our active adaptation during the Covid-19 pandemic in terms of both a psychological acceptance of online interviews as an approach for data collection as well as our behavioral adjustment and negotiation. Through proactive prediction of upcoming situations and reflections on the past, we reconfigured our ways of doing research to achieve the goals of collecting rich data in a more efficient and ethical manner while transforming our roles as ECRs.

Finally, the concept of achievement, developed from Sen’s (1985) notion of “functioning” to denote the ways of “being and doing” valued by different individuals, refers to the transformative outcome of resources and agency, explained earlier, where individuals have more opportunities to make meaningful choices (Kabeer, 1999a). In this study, changes in resources, for example, from offline to online, may affect how we see ourselves as researchers and the ways we do our research. While agency is more related to how we set our goals and act on them, achievement emphasizes the “functioning” or new perspectives and practices for being researchers and conducting our research. In this study, the achievement dimension is seen through our understanding of online data collection experiences, including how we perceived its benefits and limitations, and our ability to make meaningful ethical choices through the adoption of online interviews during and after Covid-19.

## Method

In this study, we incorporated collective autoethnography to understand ECRs’ experiences of doing and understanding research during and after the Covid-19 pandemic. Through this approach, we created insiders’ accounts of how we, with shared identities as ECRs, coped with the changes and challenges of conducting research, the application of online interview methods, and the subsequent changes in our research practice it brought about, to understand the new operationalizations of online methodologies in academia during and after the pandemic. This collective autoethnographic approach allowed us to humanize and examine the processes and outcomes of our stories as insiders ethically (Lapadat, 2017) and delve into our “multiple layers of consciousness” (Ellis & Bochner, 2000, p. 739) to scrutinize “transient, nuanced and complicated lived experiences” (Li et al., 2023, p. 6). In this case, autoethnographic narratives help channelize ECRs’ voices so that other insiders (cultural members, e.g., participants, supervisors, and universities) and outsiders (cultural strangers, e.g., the wider academia) can capture a better understanding of our positioning (Ellis et al., 2011).

This autoethnographic study is understood as a critical interrogation of the first three authors’ experiences and perceptions. It was conducted by the first three authors, who were all PhD students studying at an Australian university. All three authors were doing educational research through qualitative methodology, seeing participants as a valuable source of information and contributor of the knowledge construction. Therefore, in all studies, interviews (individual or group) were the main method of data collection. All three authors conducted both online interviews and offline interviews depending on the situation of the Covid-19 pandemic and the choices of the research, as we will further elaborate later.

Author 1 is a third-year PhD student who was investigating English teaching and learning in China’s higher education. Due to the relapse of the pandemic, she first conducted offline interviews and then transferred to the online mode. Author 2, also from China, is a third-year PhD. Her research involved interviews and focus groups with stakeholders in the school contexts. In addition, she also investigated international PhD students’ experiences where she interviewed PhD students from six countries individually through Zoom at the time of writing this article. Author 3, a second-year PhD student, is from Indonesia, and is interested in discourse analysis, assessment and technology in education. Before starting her PhD study in 2021, she collaborated with her colleague in conducting research on an online academic reading circle through the lens of Community of Inquiry. For her PhD study, she is investigating moral and ethical discourses in technology-mediated assessment in Indonesian private universities where she allows participants to choose in person or online interviews.

The data of this study were collected through the three authors’ writing of personal retrospective narratives of their experiences, feelings, and influences during the Covid-19 pandemic. Notably, the narratives were guided by: (a) How did we perceive the changes and challenges in data collection during the Covid-19 pandemic? (b) How did we understand and ethically negotiate the changes during online data collection? (c) How does such negotiation influence our future research methods after the Covid-19 pandemic?

Based on these questions, the authors created their preliminary autoethnographic narratives commencing in July 2022. The three authors then read and cross-examined all the narrative reflections, and based on the questions, asked critical questions such as what the responses of the participants were, and how the change in data collection mode influenced the richness of the data. Also, the three concepts in empowerment theory were integrated into the new questions and more details and descriptions of feelings and events were added. By this means, two rounds of data collection would provide a thick and raw allocation of their experiences and personal knowledge. The data were then analyzed, following the procedures of familiarizing data recursively, identifying and highlighting keywords and common words, searching themes and final reviewing (Creswell, 2009). We created a list of keywords and codes based on the predetermined concepts and inductive key points. After a few rounds of discussion of modifying the repetitive codes, summarizing and connecting the codes, naming and rephrasing the themes were further categorized, connected, and analyzed through the empowerment framework described above.

## Findings and Discussion

The following section presents the analysis of the narratives by three researchers. We lay out our stories through the lens of the three concepts of empowerment from Kabeer: resources, agency, and achievement, as discussed above.

### Resources: (Dis)empowerment of Researchers During the Pandemic

A key aspect of *power* is the ability to make choices which “necessarily implies alternatives” (Kabeer, 1999a, p. 2). However, the turmoil caused by the Covid-19 pandemic limited the range of research method “alternatives” that were previously available for the authors but the goal of conducting ethical research and rich data remained unchanged. In that sense, researchers, particularly those who had less experience in doing research, often felt disempowered by constraints imposed by the pandemic. The authors attempted to empower themselves by adjusting their data collection methods so that they could ethically conduct the research while obtaining rich data. We considered all the resources available or unavailable for the authors at the time, then exercised our agency to inventory all available resources. Six kinds of resources are identified and analyzed based on the authors’ narratives.

First, the availability of instructional resources in the form of methodology books and articles specifically about online interviews were scarce. Such research handbooks are important resources to inform and thus enable researchers, novice researchers in particular, to exercise choice. As discussed above, the offline mode was seen as the norm for interviews before the pandemic, which was manifested in the deficient discussion on online modes in research handbooks. For instance, Braun and Clarke (2013) only introduce online textual interviews in their book, while Bryman (2016) views online video interviews as the same with its offline counterpart. Gray (2014) and Creswell and Poth (2018) consider video interviews as an efficient alternative to the offline mode, albeit in a short discussion. Most hands-on instructions in such books are given in the offline forms tacitly, such as the suggestion from Gray (2014) about observing interviewees’ body language which is hard to implement online. Likewise, discussions on ethics for qualitative research in these books also center around offline interviews (see, for example, Bryman, 2016).

As a consequence of such limited access to instructional resources, many ECRs were confronted with the research methods which they had not trained or prepared for, experienced in, or even considered doing until the sudden outbreak of the pandemic (Howlett, 2021). This may explain why A2 narrated: “Although there were possibilities of conducting online interviews and focus groups (which are the main methods in my study), I did not hesitate to travel all the way back to China.” She tended to choose the methods with which she was more familiar. Unexpectedly, A2’s insistence on offline interviews considerably slowed down her research, which will be analyzed below.

Second, due to such lack of instructional resources, the authors could only rely on their personal experiences of conducting online interviews. While A3 had the experience of teaching on Zoom, thereby acquiring the strategies of building up rapport and organizing the online focus group effectively, A1 was more powerless as a novice researcher without any relevant experience when she faced issues which were not discussed in the handbooks. Taking Gray’s (2014) suggestion about observing interviewees as an example, in online interviews, many participants chose to turn off their cameras, which hindered the authors’ observations of metalinguistic features of the interaction. However, until composing the narratives for this research, A1 did not realize she could encourage her participants to message her privately during the Zoom focus group, which is an advantage of online interviews and impossible in the offline mode. While this could potentially produce more unexpected data, it was not achieved obviously because of A1’s insufficient experience. We believe this would have been a fairly common experience for ECRs during the pandemic.

Third, other than the instructions, access to the internet and digital applications served as a form of resource in the authors’ experiences. In Australia and China, all teaching was delivered via video calling applications during the pandemic, and therefore all participants of A1 and A2 had access to the internet and Zoom as organizational users. In A3’s case, nevertheless, she and her colleague needed to provide compensation for the mobile data used by her participants on grounds of the limited access to the internet in Indonesia, which generated extra expenses for the participants. The limited functions of Zoom personal accounts also disempowered her to implement online interviews as intended:

As we were using the basic Zoom application, we had to re-login three times which can be time consuming. The limitation may interrupt the on-going discussion as we often needed to stop in the middle of discussion, be more aware of the 40-minute time limit and continue the discussion after re-joining the Zoom meeting.

Thunberg and Arnell (2021) point out that while online modes could save travel time and costs for research, the differences in the types of video interview applications available for researchers may produce extra costs. Financial support is therefore a necessary resource to empower researchers, which will be discussed later in this section.

Fourth, as a form of social resource, researchers’ relationship with the participants is encompassed in the definition of resources (Kabeer, 1999a). The participants of A2 were international PhD students who were familiar with interviews, and thus they actively took part in the interviews. By comparison, A1 found it hard to engage with her participants for several reasons. First of all, most of her participants were undergraduate students who had no prior experience of being interviewed for a research study. Besides, A1 had never known her participants before, and they “did not have opportunities to build up rapport because of the pandemic restrictions.” Moreover, there was a lack of instructions on and experience of doing online interviews for A1, despite some very recent articles providing suggestions on rapport building *after* A1 conducted her research (e.g., Khan & MacEachen, 2022; Żadkowska et al., 2022). As a result, A1 reported difficulties in engaging with and encouraging her participants.

Fifth, local restriction policies were observed as a determinant in either enabling or disabling the authors in making choices. For example, A1 had no choice other than online interviews on grounds of the strict lockdown when she was planning to collect data in Shanghai. For this reason, she quickly changed her plan. However, also in the Chinese context, A2’s experience was fraught with twists and turns as the restriction policies had changed, often abruptly, several times. Under such an uncertain situation, A2 decided to await the easing of restrictions, which however slowed down her research and triggered other problems:

Another unexpected challenge resulting from the Covid-19 is that because I came back from Australia, where the Covid-19 situation was much more severe than that in China, back then, some teachers/school leaders rejected my invitation to participate in research due to concerns about public health. They were worried that I might be infectious and could not risk any chance of infecting the students.

At the time of writing this article, although travel restrictions triggered by the Covid-19 pandemic had been lifted in many areas, researchers still needed to carefully deal with potential health risks (Arya & Henn, 2021), as a way of protecting their participants from potential harm. There is also some uncertainty about the future given that several infectious diseases, such as monkeypox, have been emerging worldwide, with all schools closed in Uganda due to an outbreak of Ebola. Under these circumstances, much more of people’s lives were still “being lived online” than during the pre-pandemic era (Howlett, 2021, p. 399). Researchers needed to be aware that sitting and waiting for uncertain offline research opportunities would potentially disempower them for some time, as it was still unlikely for life to resume to the pre-pandemic normal. In this article, we thus emphasize the need for researchers to generate and adjust to new understandings of social science research methods in the post-pandemic world.

Sixth, an economic resource which is often imperative in deciding data collection methods is research grants. During the pandemic, international travel grants were suspended by our university, which meant considerable research expenses had to be defrayed personally—which in the case of A2 was self-funded. In contrast, A1, despite being in China, collected her data online, which was much more affordable. It is predicted that Australian universities would suffer an annual revenue drop of 20% to 24% in the years to come (Tucker & Batagol, 2022).

It is likely that universities in other areas are experiencing the same situation under the influence of the social and economic ruptures which have arisen in the aftermath of the pandemic. Budget shortfalls and funding cuts would thus disempower researchers to a large degree, in which case the choice of online interviews could be seen as an alternative means to researcher empowerment. However, although online data collection may be less expensive due to the cut of travel expenses and logistics, we believe guidelines related to research grants for online data collection should be further developed, adjusted, and established to better support research expenses. The negotiation of these six resources, explained in this section, has further shaped how the three researchers reflect, adjust, and project their studies.

### Practising Agency: Reflecting, Adjusting, and Projecting

The second theme that was commonly found in our narratives is related to agency, practiced throughout the experiences of integrating technology in undertaking our research. Here, by synthesizing the conceptualizations of agency by Kabeer (1999a, 1999b, 2005) and Emirbayer and Mische (1998), as explained above, we understand agency as a process in which individuals *act upon* their goals by engaging *past* experiences (in this case, online teaching) and repertoires (in this case, rules and norms of doing educational research) reflexively, adjusting actions to the *exigencies of emergencies*, and *projecting* development and goal achievement in the future. These three temporal dimensions of agency weave into the efficiency in performing and transforming our roles as ECRs to compensate for the deficiency of the resources and our disempowered positioning during the crisis.

The lack of resources before, during, and after the outbreak of the Covid-19 pandemic, as presented in the first section, necessitated transformative actions to achieve our shared goal of generating rich data. Such a transformation, however, did not naturally happen; it inevitably involved our reflexivity of past experiences, our evaluation and constant negotiation of the existing and lack of resources, and capacities within the emergent conditions. A2 and A3 reflected on and learnt from the potential problems and benefits manifested in the previous adoption of online media for communication or teaching:

Our (my colleague and I) familiarity with digital technologies in online classes showed us the possibility of collecting data through apps such as Google forms and Zoom, although I personally missed the natural human interactions. But then, looking back, as we had the meetings at home, we didn’t have to spend time driving from home to campus, finding a place to discuss the meeting and ordering food. (A3)

My doubts about online data collection mainly came from my experiences attending and giving online classes, where interaction was not as smooth as in offline classrooms. (A2)

These narratives resonate with Abidin and de Seta’s (2020) argument that online methods naturally cause doubts and tensions, considering the difficulties and worries about generating information-rich conversations with participants (as explained by A2). However, Abidin and de Seta (2020) seemed to have overlooked that the influence of researchers’ successful past technological integration (discussed as personal experiences in the previous section) could afford the strengthening of the perceived ease of use and usefulness of transition. A case in point is the functions of online applications which can supplement the unavailability of offline communication—as manifested in our experiences.

It seems that this more critical reflection and reflexivity, looking at both positive and negative sides of technological use, reveals a more complicated and multifaceted inner struggle that affects researchers’ decision-making. The sudden transition to online data collection was primarily triggered by the emergent situations during the Covid-19 pandemic, when the online approach was a necessity (Li, 2022; Howlett, 2021) rather than a choice. Our previous adoption of technology in other scenarios (such as teaching and social networking, indicated above) informed the following preparation of online interviews and focus groups. For example, we transferred the previous technological integration in teaching and social networking into mending the discrepancies between offline data collection and its online counterparts by eliciting questions to everyone to guarantee the participation of all interviewees so that participants would be more likely to feel respected and engaged. Such agentic action based upon previous patterns of behavior created projective modifications of our new attempt to interview participants online (Emirbayer & Mische, 1998). Despite such preparation not preventing all the problems that we met in our online data collection, it somehow alleviated the potential threats of collecting rich and sufficient data. These attempts manifest the exercise of effective agency to efficiently collect data in a time of crisis. As A2 wrote,

For example, we used the transcription function in Zoom to record the conversations, saving a lot of time typing and transcribing. Also, it is more natural to do a video recording of people’s expressions and gestures on Zoom, compared with the often audio-only recording in many offline interviews/focus groups.

The reflection on the experience of using online apps or platforms in other genres bridged our transition to online data collection and helped us adapt to a different structural environment during a crisis at that time. It also provided more well-thought-out rationales for online data collection, not only in relation to the Covid-19 pandemic but also to reveal our stronger determination and an exercise of personal choices:

After considering such factors, I collected the data online and started a new-fangled but tortuous experience. If I wanted, I could only collect data in Yunnan in person as half of the data was enough. I could also wait until the restrictions in Shanghai were eased. But I decided to do it online because I believe I could collect enough data online. (A1)

It is in this sense that our experiences demonstrate the “practice-evaluative element” of agency (Emirbayer & Mische, 1998), in which we developed higher capacities (or acquisition and use of new resources) to make practical judgments of possible trajectories of action (online or offline data collection). It also changed our conventional mindset of seeing “offline data collection as granted” (A2) and foregrounded the negotiation of possible outcomes of different modes of data collection.

In addition, the *acting upon* emergency was not limited to the process of conducting a study in an emergency—as perhaps regularly expected of PhD students—but also permeated to our further contemplations of the approach of undertaking future research. Talking about this issue, A2 explained,

I think there are differences in the reasons why I want to do offline interviews between now and then: instead of simply reckoning the inefficiency of online interviews and focus groups, it is more about weighing the benefits and drawbacks of two ways of data collection with less bias beforehand. I now see online data collection as a solid option to go with in data collection rather than a Plan B.

This experience led to the transformation of our researcher roles (Al-Salom & Miller, 2017), helping us to obtain a “power within” (Kabeer, 2005, p. 438) and having “more options in collecting data for my PhD” (A3), which indicates our empowerment, as presented in the next section.

### Achievement: Understanding Ethical Ways to Co-Construct Meaning

Resources and agency, discussed earlier in this study, constitute “the potential that people have for living the lives they want, of achieving valued ways of being and doing,” and achievement is the extent to which such potential is realized (Kabeer, 1999b, p. 438). For researchers, therefore, resources and agency make up the potential for doing high-quality research in a rigorous, ethical, and meaningful manner. Accordingly, achievement refers to the extent to which this potential is realized. Under this aspect, we identified three kinds of achievements from the authors’ experiences of adopting online interviews during the pandemic—gathering sufficient data, negotiating new ways of conducting research, and developing a new understanding of research ethics in practice by discussing the methodological choice with participants.

The three authors collected adequate data through online interviews, although their satisfaction varied. Providing rich and informative data for research is a characteristic of interviews (Barrett & Twycross, 2018), and thus it is the major purpose and achievement of doing research. A2 interviewed international students with similar backgrounds and experiences, which made the participants somewhat able to “express themselves effectively.” Therefore, she expressed surprise about “how rich data the online interviews generated via Zoom,” thereby believing that online interviews could be as efficient as offline interviews in terms of co-constructing knowledge, which accords with the findings of Thunberg and Arnell (2021). Similarly, since A3 had prior experience of online teaching, she explained that “gathering data through digital technologies provides some convenience.” She encouraged her participants to express themselves freely, thereby receiving articulated responses from her participants. Nevertheless, A1 was not satisfied with the richness of the data she collected online: “I collected more data online than I thought, but still not as rich as face to face due to the constraints mentioned above which I could overcome if I had experiences or instructions.”

She clarified that she did not know anything or even consider online interviews before the local outbreak of the pandemic which suddenly forced her to transition from offline to online interviews. This provides evidence to the opinion expressed by Howlett (2021) and reiterates the importance of instructional resources for ECRs as discussed previously. A1 also conceded that she could have taken advantage of online interviewing applications, such as the private message function mentioned above, to obtain richer and deeper data. A similar conclusion was reached by Wray and Barrett (2022) who argues online interviews are able to collect rich and varied data *when* used appropriately and effectively. Such experiences encourage the authors to reflect on our research projects and gain perspectives on how to use online interviews to overcome some of the research challenges, socio-spatial and health-related challenges in particular. This leads to the second achievement—negotiating new ways of implementing research.

One aspect of this negotiation lies in the authors’ increasing acceptance of technology in conducting research during their online data collection. A2, for instance, was initially skeptical about online data collection but gradually changed her opinion:

The most important implication I draw from such experience is that I gradually understand the process of online data collection and accept it. I now see online data collection as a solid option to go with in data collection rather than a Plan B.

Wray and Barrett (2022) argues against a “better-to-worse” hierarchy of data collection methods before the pandemic which viewed online interviews simply as “better than nothing” (p. 45). The varying opinion of A2 refutes such a hierarchy and supports the argument of Wray and Barrett (2022) that online interviews should be on an equal footing with offline interviews instead of a last resort when the latter is not available. A3’s narrative further points to the strengths of online interviews in comparison with the offline method: “We had the discussion from our own convenient space which makes it relaxing for me to ask questions. One student from a different part of the country joined the discussion while having lunch.”

Aligned with the study of Schulte-Römer and Gesing (2022) who suggest that online interviews are easier as no physical presence is required, this data collection method enabled the authors to engage with distant participants. Online interviews also give the participants more flexibility in deciding their time and space, which will be discussed further below. On the other hand, it is more difficult for researchers to manage the participants’ attention, illustrated by the participant who was having lunch during the interview. This indicates methodological and ethical challenges brought by online interviews.

Having accepted the use of online methods in interviewing, the authors also negotiated new ways to address the new challenges emerging in online interviews. Both A1 and A3 reported difficulties in making small talks, which they considered important in building rapport with participants. Located in different areas with her participants, A1 found it hard to find a “common topic” for a small talk. A3 initiated but hardly maintained the small talks due to the lack of instant responses from the participants. Although online conversations appeared more organized, they tended to be less “natural” as the participants often kept their mic on mute or the camera turned off. A3 expected more spontaneous interruptions during conversations to enrich data, whereas most participants were muted when others were talking. After interviewing, A1 reflected on the ways to encourage interaction between participants: “I would also encourage them to use the chat box function of Zoom to comment on and interact with each other. Talking simultaneously is impossible online because it makes noise, but textual chatting may be appealing to students.” Although verbal communication and nonverbal information, such as facial expression and body language, are limited in online interviews, the authors explored a new way to prompt interaction by using textual chatting.

As Newman et al. (2021) suggest, online data collection methods require more careful consideration of the methodology and ethics. Along with the methodological change, researchers need to update their understanding of doing research more ethically. With a higher acceptance of online interviews, seeing them as a normal mode of data collection, new understandings of ethical, responsible, and respectful conduct of research are developed in the authors’ practice, which is the third aspect of their achievement. Before transitioning to online interviews, the authors had been aware of how to address issues of procedural ethics (Guillemin & Gillam, 2004), the routinely ethical issues such as building good relationships with online participants, clear and transparent descriptions of the research aim, procedures, risks, and benefits, which had been considered before commencing data collection. Protocols of conduct, as required and approved by the university’s Ethics Committee, were strictly followed by the authors as a routine way of doing ethical research. When negotiating the choice of online or offline modes with the participants, nevertheless, the authors were confronted with unexpected ethical issues. While A3 and A2 gave the choice to their participants, A1 was provided by her participants with the options. After analyzing the resources available or unavailable, the authors exercised their agency and finally made ethical decisions in ethically important moments.

Having previously achieved positive online interview outcomes, A3 decided to offer an online/offline option to her participants. She believed that providing online options for the participants could help to accommodate to her participants’ schedules and their health demands. For some of her participants, online options were the preferred mode due to practicality and proximity—some were not in the city at the time of interview, whereas others had to manage teaching and administrative agendas. For this reason, A3 did not need to wait for a long time. In addition, one of her research participants chose the online option as they were feeling under the weather. Similarly, A2 also offered both online and offline options to the participants in her doctoral research, which investigated late-career teachers’ lived experiences. However, unlike A3’s experiences, her participants were more inclined to offline interviews, due to their wish to show their working environment to the researcher as well as what researchers call screen fatigue (Pandya & Lodha, 2021). A2 mentioned that one of her participants said, “I would be more engaged in an offline conversation, and I could show you my classroom, my students, and the everyday work, so that you could have a better understanding of our normal days.” Although A2 had to wait longer for the availability of her participants, she gathered rich and high-quality data.

In these two cases, providing options to the participants helps to address their health concerns—the risk of getting sicker and screen fatigue. Moreover, the participants made the choice to provide data in the ways perceived by them as effective—a short wait for A3 and close contact for A2. This indicates that the empowerment was not limited to researchers, but also participants who are invited in the decision-making process. As Guillemin and Gillam (2004) point out, many studies see participants as “subjects,” which means they are used merely as means or tools (p. 271). Only when researchers acknowledge the autonomy of participants as decision makers in their life, they become “participants” (Guillemin & Gillam, 2004, p. 271). At the procedural ethics level, this principle is reflected as informed consent (Guillemin & Gillam, 2004). A3 and A2 achieved this principle at the ethics in practice level in the form of giving the choice to the participants, thereby showing the researchers’ respect for the autonomy of the participants and invited them to the decision-making procedure in the research. The participants’ selection in turn empowered the authors through the provision of richer data.

The experience of A1 is quite different. In the face of the lockdown, her participants offered her an option—sneaking into the campus. In this case, the moment of A1 negotiating with her participants serves as an ethically important moment where the decision made by the researcher has important ethical results (Guillemin & Gillam, 2004). If A1 insisted on offline interviews, she might have successfully collected enough data, but unethically. This would put A1 and her participants at potential health risk and made her fail in maintaining a respectful relationship with her participants. A1 evaluated all the resources she had and weighed up the risks and benefits through an exercise of agency, then identified online interviews as the only ethical option at that moment. By discussing and making decisions with her participants, A1 showed respect for the participants’ health and safety and built up mutual trust with her participants. Afterward, she drew on available resources and exerted her agency—as discussed in the previous sections—and finally obtained rich data. In this case, the availability of online interviews empowered A1 from “no other ethical choice” to be “able to finish data collection ethically,” and her negotiation with her participants on the choice of online interviews achieved ethics in practice.

While discussions on online interviews often center on privacy issues (see, for example, Newman et al., 2021), negotiating with participants on the choice enables them to have control over to what extent they would like to share their space with the researcher. Researchers can also build up a more respectful relationship with the participants, by sharing the privilege of designing steps of research. This position corroborates with the principle of conducting research in an ethical manner, protecting the rights of the participants, creating a more equal and safer environment for participants to share (Creswell, 2009). Particularly, this new approach could avoid unequal power relations between researchers and participants. For example, in the case of A3, considering that some participants were her former students, she could enable them to feel in an equal status and have the power to voice their own preferences in the research. It also means that the two authors developed a higher level of flexibility and sensitivity in conducting research, giving more thoughts to their participants’ conditions and “meaningfully engaging diverse individuals” (Newman et al., 2021, p. 1).

## Final Remarks

This article has presented how three PhD researchers adjusted to online interviews during the Covid-19 pandemic and how such experiences shaped their understanding of conducting research through the empowerment framework (Kabeer, 1999a, 1999b, 2005). Together, such experiences have manifested that online interviews can be an empowering option to obtain rich data for qualitative research. It is also shown that negotiating the choice of data collection modes with participants can serve as a new aspect of ethics in practice. The narratives presented demonstrate how online interviews can bring about empowerment through the three interrelated dimensions, namely, resources, agency, and achievement.

We also reflected on how instructional, social, economic resources can facilitate or constrain researchers to exercise agency by engaging their past experiences reflexively, adjusting their choices, and projecting future research work. In the face of a host of challenges and limitations, by exercising agency, the researchers were able to recruit and build rapport with their participants, engage with the meaning making process, and gain in-depth data as well as gain a nuanced understanding of conducting online research ethically as seen in the achievements in their narratives.

Although acting upon the research goals was made possible through online data collection, we also articulate some challenges when doing online interviews. Differences of socio-emotional connection during interviews, such as those cases of turning off the camera and muting microphone, may lead to greater flexibility for the interviewees but also at the same time result in limited nonverbal expressions which may be vital for qualitative research. This, however, can be managed through online interviews noting that both researchers and interviewees build mutual understanding on the importance of gestures and nonverbal expression during online interviews. Reflecting critically on research achievement to further enhance instructional, social, and economic resources to further exercise temporal agency is thus of critical importance to provide better online research facilitation.

Despite ample evidence of a range of limitations of online interviews, they can still facilitate achievement in data collection—in terms of gaining better perspectives of online interviews, demonstrating technology acceptance and negotiating an ethical way of doing research. In this sense, understanding and accepting online data collection mode as a solid way of doing research during and after the Covid-19 pandemic can enable researchers to negotiate ethical online and offline alternatives for interviews. With these options, researchers are able to respect the rights of participants to choose a more equal and safe space for them to voice out their stories. We resonate with Fielding’s (2014) suggestion that it is critical to negotiate contextual resources such as equipment, software, and more online workshops in conducting online interviews in digital futures. It should be noted, then, that the research outcomes in our writing are situated within the context of the pandemic and post-pandemic where people are concerned about public health. Some other factors as discussed earlier such as the rapport with the participants, the experience and skills of the researchers and participants, the features of the digital platforms, and the nature of the research affect the end results of the data gathering processes.

The three authors’ experiences of choosing and implementing online interviews during the pandemic have also demonstrated the uniqueness of online interviews—it is neither inferior nor better than offline interviews but a unique data collection method with its own sets of benefits and drawbacks. Therefore, we argue that research institutions and researchers need to reconsider stereotypical attitudes toward online interviews and place online and offline data collection methods on an equal footing. Practical and detailed instructions which hold a fair view to online and offline methods should be made available for ECRs to have a comprehensive understanding of qualitative research methods and to pragmatically choose the one most suitable for their studies.

Furthermore, this study shows that online interviews also generate expenses, albeit often more affordable than its offline counterpart. Nonetheless, such costs are sometimes not provided by institutions which can disempower researchers. It is suggested that research institutions should not deem online interviews as free. Financial support for online interviews should be offered for more effective data collection and research outcomes.

We acknowledge the limitations of the current study in terms of its autoethnographic nature and its focus on experiences mainly during the pandemic. This study was conducted with three international PhD students’ experiences in an Australian university carrying out case studies about educational issues in two Asian countries. Caution needs to be taken in making generalizations from the findings and discussion since we know how the varied pandemic situations across regions and the diversity of research may affect researchers’ positionality, in terms of contact with participants, access to facilities, and technological readiness. Therefore, we hope this research can trigger more narratives from ECRs in different contexts (nationalities, institutions, and subjects) to obtain a more holistic and broader understanding of how the Covid-19 pandemic has shaped PhD students’ academic identities and approaches to conducting research.

In addition, while this research sheds light on the amid-pandemic and immediate post-pandemic period, when the new normalcy has gradually formed after the pandemic, we expect further discussions on the lingering effects of technological-based methodology in the post-pandemic era. It is equally of importance for qualitative researchers to think beyond the practicalities of the virtual data collection, and to consider its continued functions to mediate fruitful and ethically informed interactions between participants and researchers.
